# Supplementary material for: Scale law of complex deformation transitions of nanotwins in stainless steel
Source: Nat Commun. 2019 Mar 29;10:1403. doi: 10.1038/s41467-019-09360-1 (PMC6440981; doi:10.1038/s41467-019-09360-1)
Supplement: Supplementary file 1 — Supplementary Information [file 41467_2019_9360_MOESM1_ESM.pdf]

Supplementary Information

Scale law of complex deformation transitions of nanotwins in stainless steel

Chen et al.

Supplementary Note 1

The detailed microstructure of the intersected nanotwins (NTs) are shown in Supplementary Figure 1, where the intersected angle of the two NT systems is 70° (Supplementary Figure 1a). The corresponding selected-area electron diffraction (SAED) pattern confirms the formation of NTs (Supplementary Figure 1b). The dark-field TEM image (Supplementary Figure 1c) clearly shows that the twin-lamella-spacing (λ) of NTs are in the range of 1.0 - 4.0 nm. The high-resolution TEM (HRTEM) image (Supplementary Figure 1d) displays the NTed microstructure of the rectangular zone in Supplementary Figure 1a. A large number of dislocations, marked by "$\top$", are formed close to the twin boundaries (TBs). The λ of these NTs are from 0.95 to 4.37 nm.

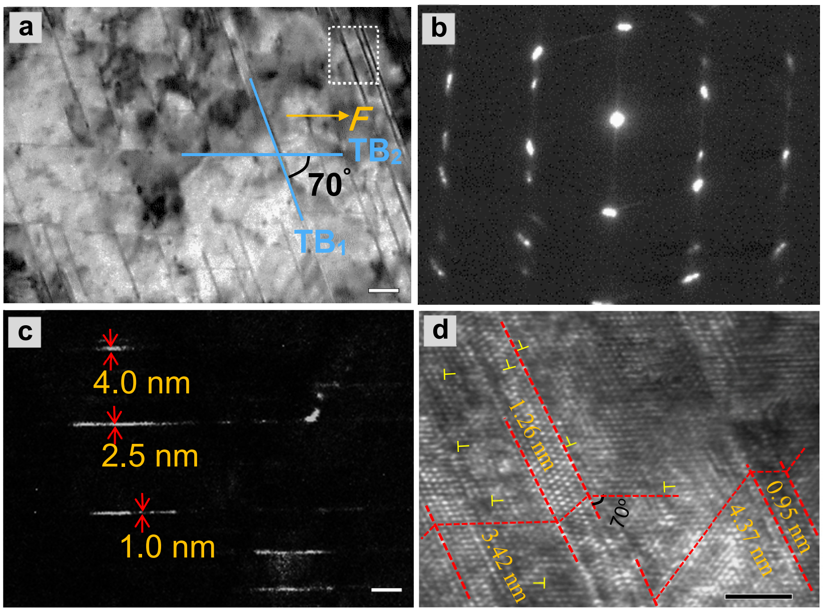

Supplementary Figure 1 Microstructure of the intersected NTs. **a** Bright-field TEM image, showing the microstructure of the intersected NTs. Scale bar is 20 nm. *F* represents the external tensile load. **b** SAED of **a**. **c** Dark-field TEM image of **a**, where the λ of these NTs are in the range of 1.0 - 4.0 nm. Scale bar is 20 nm. **d** HRTEM image of the white box in **a**, giving the defected structures of the NTs with λ from 0.95 to 4.37 nm. Lots of dislocations, as marked by "┬", exist inside the deformation NTs. Scale bar is 2 nm.

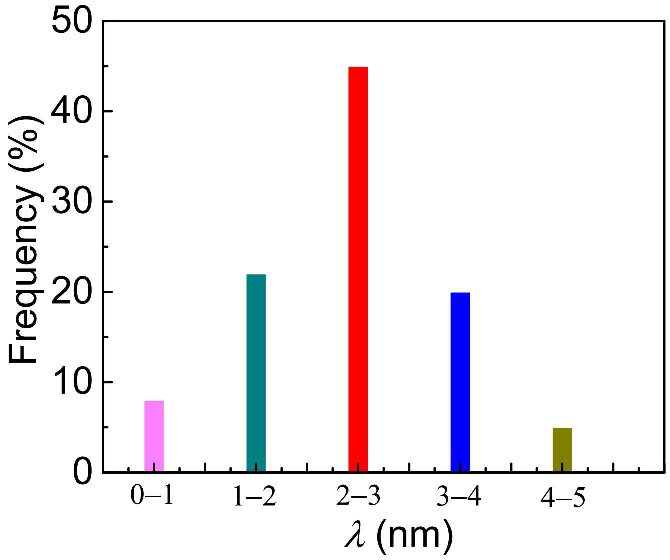

Supplementary Figure 2 Statistical distribution of λ in the intersected NTs. Coactivation of twinning and detwinning occurs in the NTs with $\lambda < 5$ nm, and the NTs with $\lambda = 2 - 3$ nm contribute the highest ratio. The standard deviation of the evaluated λ is 0.5 nm.

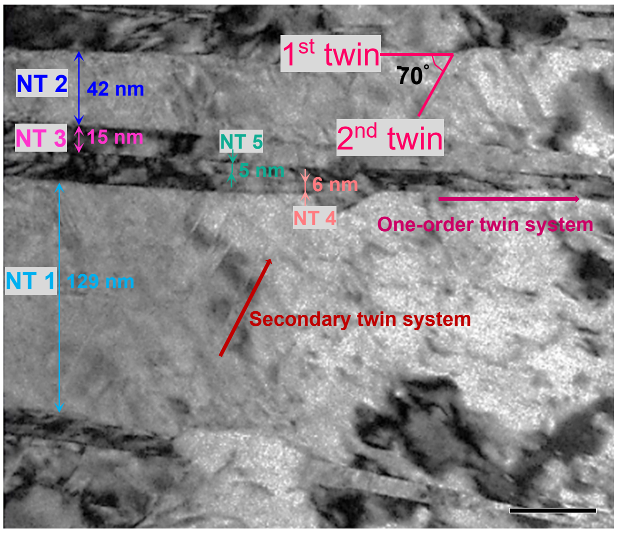

Supplementary Figure 3 Bright-field TEM of the primary and secondary twins. The λ of the primary NTs 1 - 5 are 129, 42, 15, 6, 5 nm, respectively. The 1st twin is the one-order twin system, 2nd twin is the secondary twin system, and the two systems exhibit a 70° angle. The standard deviation of the evaluated λ are 20 nm for NT 1 and 0.5 nm for NTs 2 - 5. Scale bar is 50 nm.

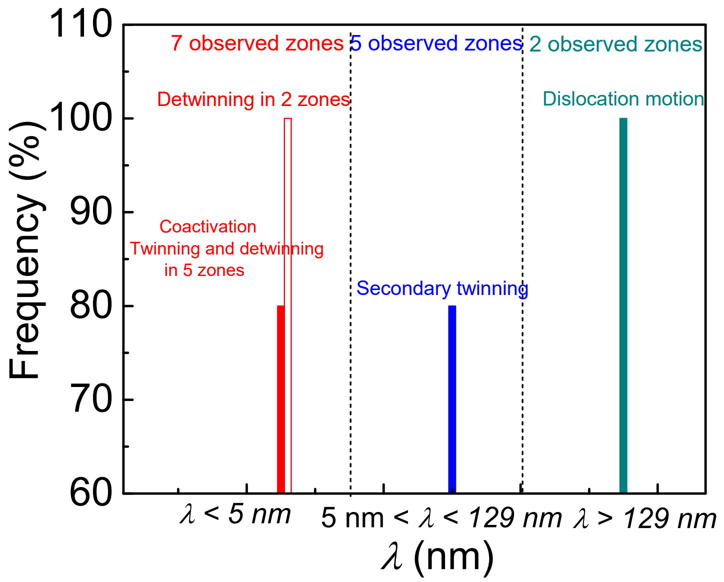

Supplementary Figure 4 The statistical diagram of the deformation behaviors. 14 microzones are observed through in situ TEM tests, where 7 observed zones contain the NTs with $\lambda < 5$ nm, 5 observed zones have the NTs with 5 nm $< \lambda <$ 129 nm, and 2 observed zones are the twins with λ at the submicrometer scale. The standard deviation of the λ is 20 nm for the upper limit of 129 nm and 0.5 nm for the lower limit of 5 nm. The statistical results exhibit that 80% of NTs (5 observed zones with $\lambda < 5$ nm) occur coactivated twinning and detwinning under a 70° TB orientation angle to loading direction. 100% of NTs (2 observed zones with $\lambda < 5$ nm) exhibit detwinning and the subsequent martensite transformation under a 9° TB orientation angle to loading direction. Among the 5 observed zones containing NTs with $\lambda = 6$ - 129 nm, secondary twinning occurs with a frequency of 80%. In the left 2 observed zones, dislocation motion is active in the NTs with λ at the submicrometer scale.

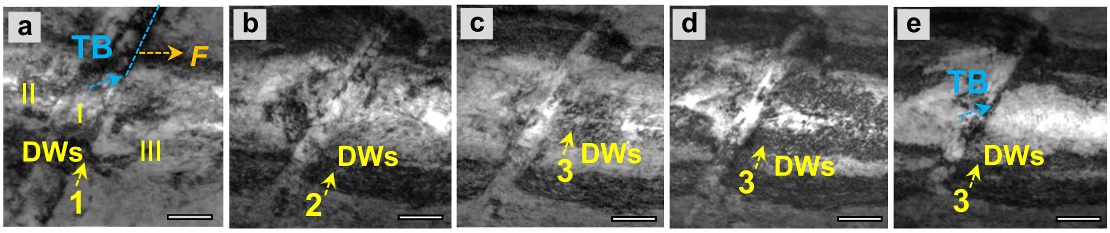

Supplementary Figure 5 The formation of dislocation walls (DWs) at the TBs. *F* is the tensile load. Three twins, I, II, III, with λ at the submicrometer scale, are observed in **a**. The dislocations accumulate at the TB and propagate normal to the loading direction in the form of DWs, as indicated by Arrow 1 in **a**. After that, a wider DW generates, as indicated by Arrow 2 in **b**. When the load further increases, new DWs generate at the TB, as indicated by Arrow 3 in **c**, and then the DWs separate into two parts, and propagate perpendicular to the loading direction in **d**. This procedure cycles, that is, the dislocations initiate from the TB, transit into DWs, and the DWs propagate perpendicular to the loading direction. The time frames from (a-e) are 0, 8, 68, 140, and 187 s, respectively. Scale bars are 200 nm.

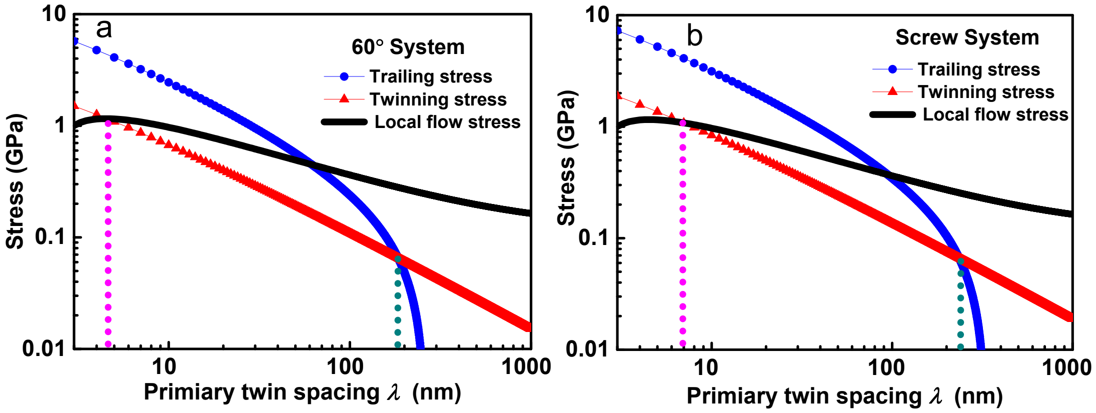

Supplementary Figure 6 The predicted critical λ for generating secondary twinning. **a** 60° system of partials under an 85.2° angle of the TB orientation to tensile direction. **b** Screw system of partials under a 26.5° angle of the TB orientation to tensile direction. The predicted range of λ for the secondary twinning is 4.8 - 186 nm for the 60° system at 85.2°, and 6.7 - 237 nm for the screw system at 26.5° through comparing the twinning stress with the local stress (upper point) and trailing stress (lower point).

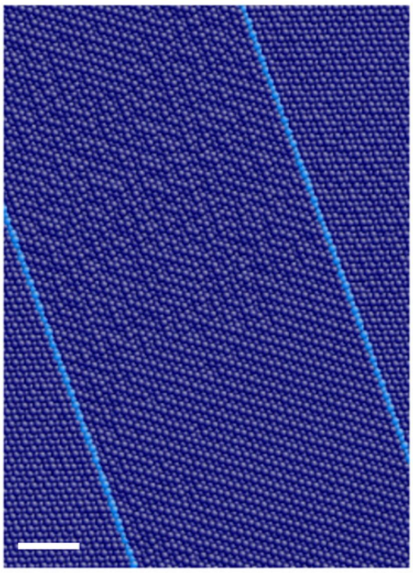

Supplementary Figure 7 Snapshot of the relaxed NT structure before tensile (λ = 10 nm). Scale bar is 2 nm.

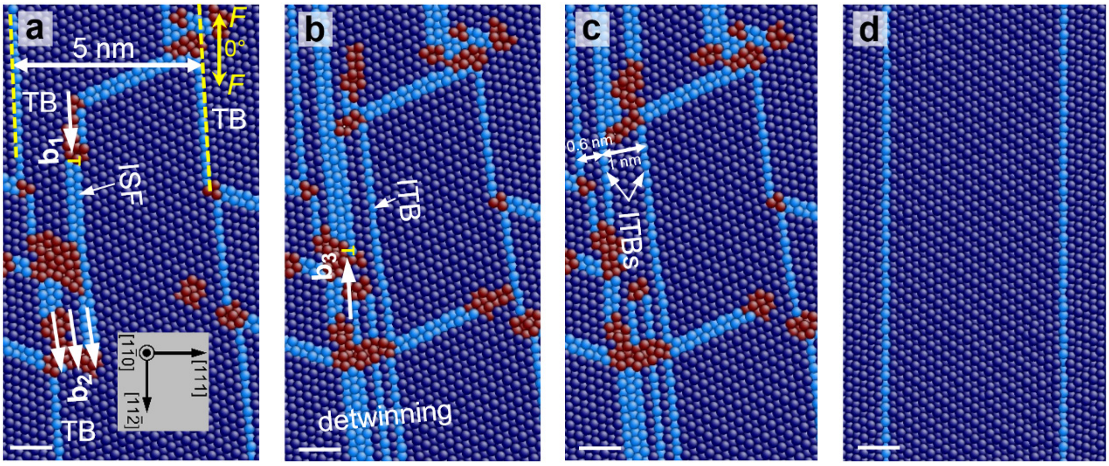

Supplementary Figure 8 Snapshots of twinning and detwinning of the TB[1st]. **a** Partial dislocations are ready to propagate. **b** The formation of incident TB (ITB) after the propagation of $\mathbf{b_1}$ along intrinsic stacking fault (ISF) and detwinning due to the propagation of a bundle of $\mathbf{b_2}$ in **a**. **c** Thickening of ITBs owing to the propagation of $\mathbf{b_3}$ in **b**. **d** Snapshot before deformation, which is the original configuration. The NT (λ = 5 nm) in Fe-Cr-Ni stainless steel (SS) is deformed under uniaxial tensile load (F) with the TB orientation parallel to the tensile direction. Scale bars are 1 nm.

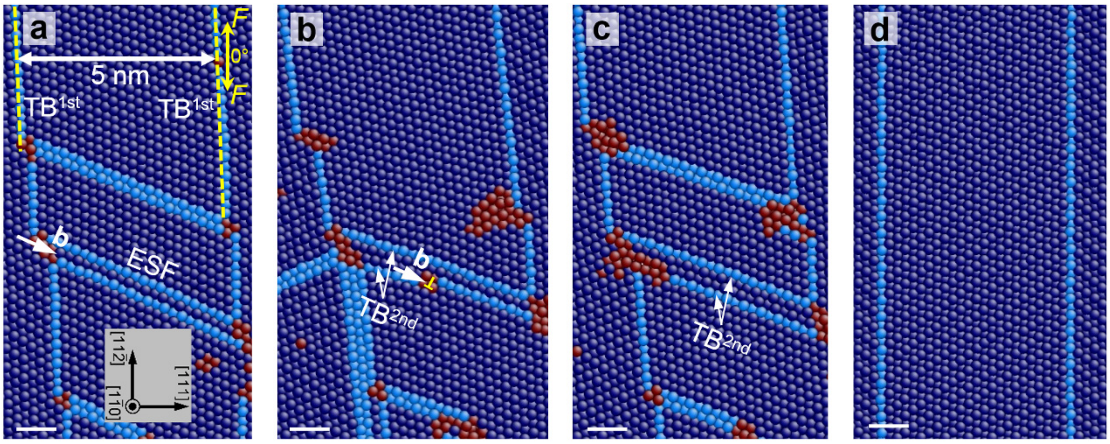

Supplementary Figure 9 Snapshots of the secondary twinning in the NT. **a** Upcoming propagation of partial dislocation **b** along the extrinsic stacking fault (ESF). **b** The formation of TBs^{2nd} along with the propagation of **b**. **c** The final configuration of TBs^{2nd} between TBs^{1st} evolved from ESF. **d** Snapshot of the relaxed configuration before deformation. The NT ($\lambda = 5$ nm) in Fe-Cr-Ni SS is deformed under uniaxial tensile load (F) with the TB orientation parallel to the tensile direction. Scale bars are 1 nm.

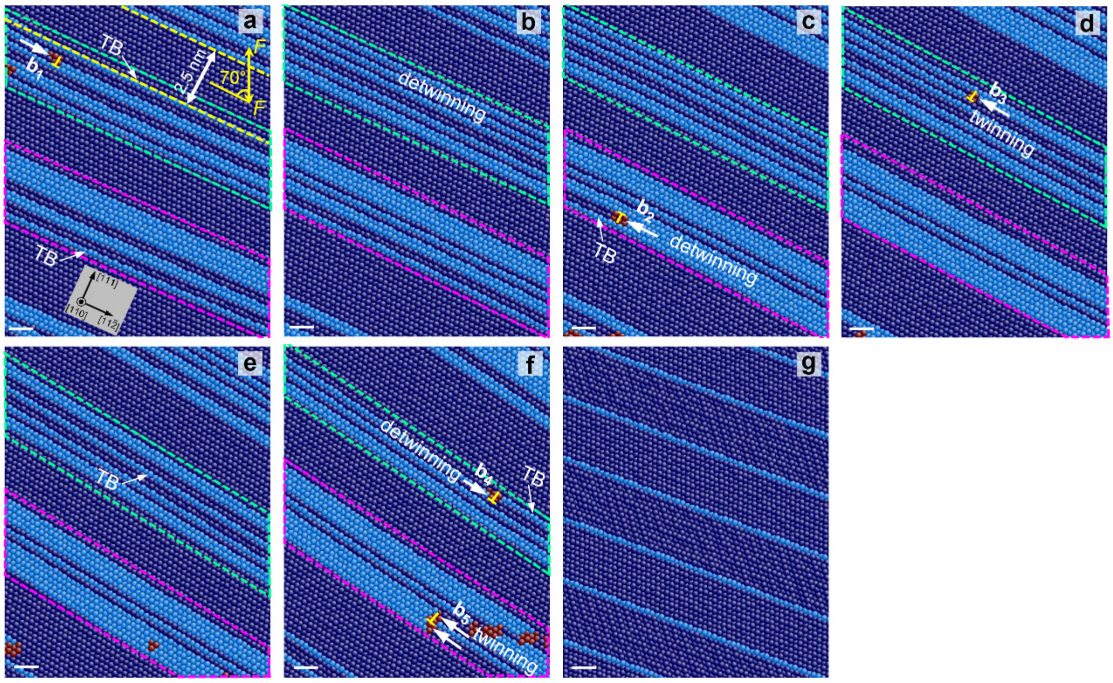

Supplementary Figure 10 Snapshots of twinning and detwinning of the NTs (λ = 2.5 nm). **a** Partial dislocation $\mathbf{b_1}$ propagates parallel to pre-existing TBs. **b** Detwinning in green region because of $\mathbf{b_1}$ in **a**. **c** The propagation of $\mathbf{b_2}$ beside the TB leads to detwinning in pink region. **d** Twinning in green region as a result of the propagation of $\mathbf{b_3}$. **e** The formation of TB in green region after the propagation of $\mathbf{b_3}$ in **d**. **f** Simultaneous detwinning and twinning in green and pink regions due to the propagation of $\mathbf{b_4}$ and $\mathbf{b_5}$, respectively. **g** Snapshot of the relaxed configuration before deformation. The NTs are deformed under uniaxial tensile load (F) with a 70° angle of the TB orientation to the tensile direction. Scale bars are 1 nm.

Supplementary Note 2 | Materials and Methods

The chemical composition of 304 SS is 0.04 C, 0.37 Mo, 0.49 Si, 1.65 Mn, 7.8 Ni, 16.8 Cr and balanced Fe (all in wt%). The NTed 304 SS was prepared by surface mechanical attrition treatment (SMAT), and the detailed processing is given in Ref[1]. After SMAT, the mean grain size of the NTed 304 SS is 15 μm. The samples for in situ tests were prepared by mechanical polishing to 150 μm depth from the surface, and then thinned also by mechanical polishing from the other side to a final thickness of 30 μm. The foils with a dimension of 5 mm × 2 mm × 30 μm were thinned by twin-jet electropolishing in ethanol solution containing 5% perchloric acid at -30 °C, as shown in Supplementary Figure 11. The in situ tensile experiment was manually controlled with an acting time of 0.5 s of each loading, and video-recorded by a Soft Imaging System Mega View III camera.

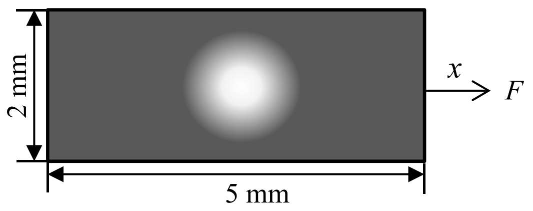

Supplementary Figure 11 A schematic picture of the in situ tensile specimen. Bright area is a thinned regime with a void in the center. The specimen are tested under a uniaxial tensile load (*F*). *x* represents the loading direction.

Supplementary Note 3 | Dislocation-based theoretical model

For the behaviors of deformation twinning in nanocrystalline face-centered cubic (FCC) metals, the analytical models based on dislocation theory have been proposed to determine the critical grain size for twinning, through comparing the critical twinning stress with the critical trailing stress[2,3]. In similarity, such analytical model could be extended to characterize the twinning and detwinning behaviors in the NTed FCC 304 SS. Supplementary Figure 12 depicts the schematic drawing of the theoretical model for nucleating a twin in a deformed region or a twin lamellae. Suppose that there is a stacking fault crossing the region surrounding by the inner boundaries such as the grain boundaries, twin boundaries or interface between two phases. The leading partial ACDB and the trailing partial AC'D'B both emit one by one from inner boundaries, and partial dislocation lines are divided by a stacking fault. As shown in Supplementary Figure 12, when $\alpha_1 = 90°$ for leading partial and $\alpha_2 = 30°$ for trailing partial, such dislocations are called as the 60° system. When $\alpha_1 = 30°$ for leading and $\alpha_2 = -30°$ for trailing partials, these dislocations are defined as the screw system[2]. According to the Thompson tetrahedron illustrating the possible slip planes in FCC crystal, the mixed dislocation and screw dislocation contribute to the deformation twinning[3]. The 60° system is associated with the mixed dislocations. Therefore, these two kinds of dislocation systems are easy to nuclear a twinning deformation in the nanocrystalline metals[4].

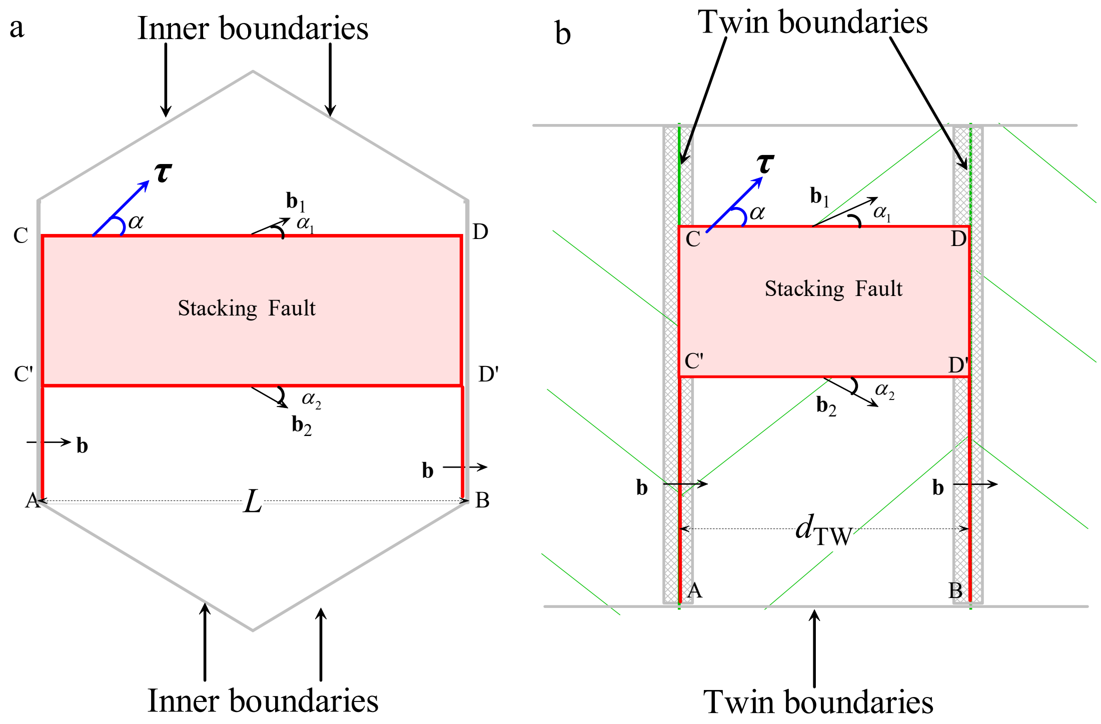

Supplementary Figure 12 Schematic drawing of the dislocation model. **a** The deformed region. **b** Twin lamellae to form a twin.

Note that the movement of leading and trailing partials as well as the stacking fault are all driven by the applied stress $\boldsymbol{\tau}$. Thereby, when CD moves a distance Δx, the work done by the applied stress can be expressed as:

$$\Delta E_{\tau} = \Delta x \cdot L \cdot \boldsymbol{\tau} \cdot \mathbf{b}_1. \quad (1)$$

From the dislocation theory, the increment of dislocation line energy can be achieved, given as:

$$\Delta E_{\mathrm{d}} = 2\Delta x \cdot \frac{G b_1^2 (1-\nu\cos^2\beta)}{4\pi(1-\nu)} \ln\left(\frac{R}{r_0}\right), \quad (2)$$

where b_1 is the absolute length of leading partial $\mathbf{b}_1$, R is approximated as the width of the defined region L, and r_0 is as the magnitude of a lattice dislocation $\mathbf{b}$. ν is the

Poisson ratio. G is the shear modulus. β is the angle between a Burgers vector and the dislocation line, such as α_1 and α_2 shown in Supplementary Figure 12. Let's define the angle to be positive when the angle rotates anticlockwise from the dislocation line. Then, the critical twinning stress τ_{twin} could be determined according to the relation of $\Delta E_\tau = \Delta E_{\mathrm{d}}$, given as:

$$\tau_{\mathrm{twin}} = C_0 \frac{Ga}{2\sqrt{6}\pi L\cos(\alpha-\alpha_0-30°)} \ln\left(\frac{\sqrt{2}L}{a}\right). \quad (3)$$

Here, $\alpha_0 = 60°$ for 60° system of dislocations, and $\alpha_0 = 0°$ for screw system. $C_0 = 1$ for 60° system of dislocations, and $C_0 = (4-v)/(1-v)$ for screw system. On the other hand, when C'D' moves a distance, Δs, the work done by the applied stress is given as:

$$\Delta E_\tau = \Delta s \cdot L \cdot \boldsymbol{\tau} \cdot \mathbf{b}_1, \quad (4)$$

and the reduction of stacking fault energy is

$$\Delta E_{\mathrm{SF}} = \Delta s \cdot L \cdot \gamma_{\mathrm{isf}}, \quad (5)$$

where γ_{isf} is the intrinsic stacking fault energy. Otherwise, the increased dislocation line energy of lattice dislocation can be derived as:

$$\Delta E_{\mathrm{d}} = 2\Delta s \cdot \frac{Gb^2}{4\pi(1-v)} \ln\left(\frac{R}{r_0}\right) - 2\Delta s \cdot \frac{Gb_2^2(1-v\cos^2\beta)}{4\pi(1-v)} \ln\left(\frac{R}{r_0}\right). \quad (6)$$

According to the energy balance between the work done by the applied stress, the energy change in the dislocation segments and stacking fault plane, the critical trailing stress can be given as:

$$\tau_{\mathrm{trail}} = \frac{1}{\cos(\alpha-\alpha_0+30°)} \left[\frac{C_1\sqrt{6}Ga}{48\pi(1-v)L} \ln\left(\frac{\sqrt{2}L}{a}\right) - \frac{\sqrt{6}\gamma_{\mathrm{isf}}}{a}\right]. \quad (7)$$

Here, $C_1 = (8-5v)$ for 60° system of dislocations, and $C_1 = (8+v)$ for screw system. Moreover, the balance between the work done by the applied stress and the

increment of dislocation line energy leads to the determination of the critical detwinning stress, given as:

$$\tau_{\mathrm{detw}} = \frac{C_2}{\cos(\alpha-\alpha_0+30°)}\frac{\sqrt{6}Ga}{48\pi(1-v)L}\ln\left(\frac{\sqrt{2}L}{a}\right). \tag{8}$$

The behavior of twinning deformation demands the critical twinning stress lower than the critical trailing stress and local stress, and the detwinning deformation occurs only when the critical detwinning stress is smaller than the local stress. Here, it should be pointed out that the 60°system and screw system are two types of classic dislocation system for deformation twinning. While the dislocation in a grain is most likely a mixed nature[2], therefore, it is difficult to separate and probe the 60° dislocation and screw dislocation in TEM tests during deformation twinning.

Another important issue is how to determine the maximum local stress in NTed metals, which must be greater than the critical twinning/detwinning stress for twinning/detwinning behaviors. A dislocation density-based plastic model has been developed in our previous work to describe the grain size and twin spacing-dependent mechanical properties of the NTed metals[5]. On the basis of this plastic model, the local flow stress in a unit of twin lamellae (Supplementary Figure 13) can be expressed as:

$$\tau_{\mathrm{flow}}^{\mathrm{L}} = \alpha_{\mathrm{T}}Gb\sqrt{\rho_{\mathrm{I}} + \rho_{\mathrm{TB}}^{\mathrm{L}}} \quad , \tag{9}$$

where α_{T} and b are the empirical constant and the Burgers constant, respectively. ρ_{I} is the dislocation density in the interior crystal, and $\rho_{\mathrm{TB}}^{\mathrm{L}}$ is local dislocation density in the twin lamellae, given as

$$\rho_{\mathrm{TB}}^{\mathrm{L}} = \frac{4\emptyset_1}{\pi}\left(\frac{2N_1}{d_{\mathrm{G}}^2} + \frac{2N_2}{d_{\mathrm{G}}d_{\mathrm{TW}}^{-1}}\right) - \frac{2\emptyset_2}{\sqrt{3}d_{\mathrm{TW}}^{-2}}, \tag{10}$$

where $\emptyset_1$ and $\emptyset_2$ are the geometric factors, N_1 and N_2 are the material parameters

independent of the twin spacing. The density of these dislocations obeys the evolution law with plastic strain as described in Kocks-Mecking model[6]. It can be found from Eqs. (3), (7), and (8) that the critical stresses of twinning, trailing and detwinning are all the functions of the orientation of applied stress α and the size of local deformation region. It means that the behaviors of twinning and detwinning in NTed metals depend on the orientation of applied stress and size of deformed region. We select the 100 - 500 nm zone as the investigation range due to the visual sight of the TEM observations. Two orientation angles of 70° and 9° between the TB orientation and the tensile direction were performed in the simulations to reveal the orientation-dependence of the applied stress on the deformation behaviors.

Since twin-spacing-dependent local stress should overcome the critical twinning stress and the critical detwinning stress, the twinning/detwinning behaviors in NTed metals are associated with twin spacing. The corresponding lower and upper bounds of critical twin spacing for generating subordinate twin lamellae can be obtained by comparing with the local flow stress, twinning stress and trailing stress, as shown in Supplementary Figure 13. Through comparing the local flow stress with the twinning stress and trailing stress, one can determine the critical twin spacing for generating secondary twin lamellae with lower bounder value. The upper bounder value of critical twin spacing for forming the subordinate twin lamellae can be obtained when the critical twinning stress is equal to the critical trailing stress. It should be emphasized that the twinning deformation occurs more easily in the 60° system since the twinning stress in the 60° system is smaller than the one in the screw system. Since the leading

and trailing partials in 60° and screw systems have various slip directions, the resulting twinning/detwinning and trailing stresses become dependent on the orientation of the partials and the applied stress. Both in the 60° and screw systems, there exits an optimal applied stress orientation for twinning deformation[3], 85.2°and 26.5°, respectively.

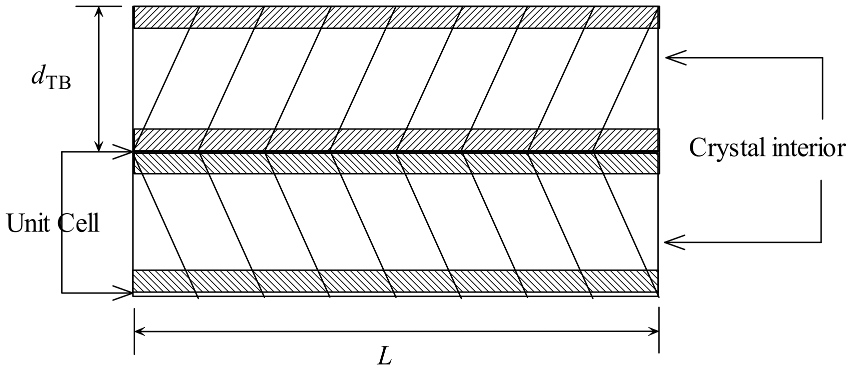

Supplementary Figure 13 A schematic of the unit twin lamellae for local flow stress.

Supplementary Note 4 | Molecular dynamics (MD) simulation

Three dimensional austenite SS samples with the composition ratio Fe = 74 at%, Ni = 8 at% and Cr = 18 at% are prepared subject to FCC structure with a lattice constant equal to 3.562 Å, as presented in Supplementary Figure 14 (colored on the basis of atom type). The model size is about 60 nm × 60 nm × 4 nm, containing approximately 1.4 million atoms. A series of such samples with different twin spacing (λ = 2.5, 3.74, 5, 10, and 15 nm) is constructed. MD simulations are carried out using the large-scale atomic/molecular massively parallel simulator (LAMMPS) code. An embedded atom method (EAM) potential for Fe-Ni-Cr system developed by Bonney et al.[7-9], which has been verified reliable to simulate the dislocation evolution of SS during plastic deformation, is employed to describe the atomic interaction in the samples. Periodic boundary condition is exerted in all three directions.

At first, the as-created samples are subjected to energy minimization by the conjugate gradient method, and then relaxed in the Nose/Hoover isobaric-isothermal ensemble under both the pressure 0 bar and 300 K for 20 ps. Then the relaxed samples are subjected to uniaxial tensile simulation along *y*-direction, strictly keeping the external pressure in the other two directions at zero. Note that two groups of samples, 0° and 70° between TB orientation and tensile direction, are simulated to compare with our experimental observations. The loading process is conducted at a constant strain rate of 2×10^8 s^{-1}. Common neighbor analysis method is employed to characterize the inherent nanostructure evolution[10].

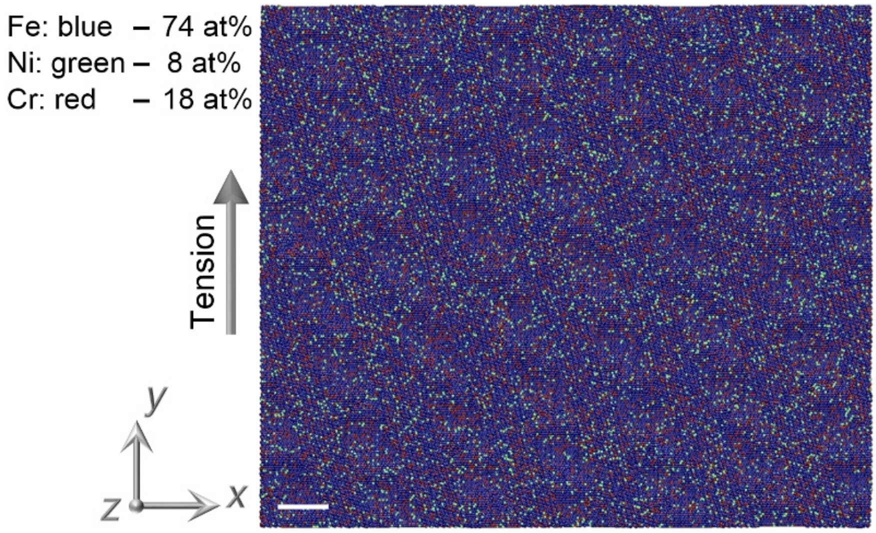

Supplementary Figure 14 The MD simulation model of austenite SS. The size is about 60 nm × 60 nm × 4 nm. All the samples are simulated under uniaxial tensile loading along *y*-direction. Scale bar is 5 nm.

Supplementary References

1. Chen, A. Y., Ruan, H. H., Wang, J., Chan, H. L., Wang, Q., Li, Q., & Lu, J. The influence of strain rate on the microstructure transition of 304 stainless steel. *Acta Mater.* **59,** 3697-3709 (2011).

2. Zhu, Y. T., Liao, X. Z., Srinivasan, S. G., & Lavernia, E. J. Nucleation of deformation twins in nanocrystalline face-centered-cubic metals processed by severe plastic deformation. *J. Appl. Phys.* **98,** 034319 (2005).

3. Zhu, Y. T., Liao, X. Z, & Wu, X. L. Deformation twinning in nanocrystalline matierals. *Prog. Mater. Sci.* **57,** 1-62 (2012).

4. Zhu, Y. T., Liao, X. Z., Srinivasan, S. G., Zhao, Y. H., Baskes, M. I., Zhou, F., & Lavernia, E. J. Nucleation and growth of deformation twins in nanocrystalline aluminum. *Appl. Phys. Lett.* **88,** 5049-5051 (2004).

5. Zhu, L. L., Ruan, H. H., Li, X. Y., Dao, M., Gao, H. J., & Lu, J. Modeling grain size dependent optimal twin spacing for achieving ultimate high strength and related high ductility in nanotwinned metals. *Acta Mater.* **59,** 5544-5557 (2011).

6. Kocks, U. F., & Mecking, H. The physics and phenomenology of strain hardening. *Prog. Mater. Sci.* **48,** 171-273 (2003).

7. Bonney, G., Terentyev, D., Pasianot, R. C., Poncé, S., & Bakaev, A. Interatomic potential to study plasticity in stainless steels: the FeNiCr model alloy. *Modelling Simul. Mater. Sci. Eng.* **19,** 085008 (2011).

8. Chen, T., Tan, L., Lu, Z., &Xu, H. The effect of grain orientation on nanoindentation behavior of model austenitic alloy Fe-20Cr-25Ni. *Acta Mater.* **138,** 83-91 (2017).

9. Terentyev, D. &J., Bakaev, A. Interaction of a screw dislocation with Frank loops in Fe-10Ni-

20Cr alloy. *J. Nucl. Mater.* **442,** 208-217 (2013).

10. Honeycutt, J. D., & Andersen, H. C. Molecular dynamics study of melting and freezing of small Lennard-Jones clusters. *J. Phys. Chem.* **91,** 4950-4963 (1987).
