# Supplementary material for: Scale law of complex deformation transitions of nanotwins in stainless steel
Source: Nat Commun. 2019 Mar 29;10:1403. doi: 10.1038/s41467-019-09360-1 (PMC6440981; doi:10.1038/s41467-019-09360-1)
Supplement: Supplementary file 3 — Description of Additional Supplementary Files [file 41467_2019_9360_MOESM3_ESM.pdf]

Description of Additional Supplementary Files

File Name: Supplementary Movie 1
Description: In-situ observation of successive twinning and detwinning in the intersected nanotwins under shear stress.

File Name: Supplementary Movie 2
Description: In-situ observation of detwinning and martensitic transformation of the nanotwins under axial stress.

File Name: Supplementary Movie 3
Description: In-situ observation of secondary twinning inside the primary nanotwins.

File Name: Supplementary Movie 4
Description: Molecular dynamics simulation of twinning and detwinning behaviors of nanotwins under uniaxial tensile deformation.
